# Automated planning of stereotactic spine re-irradiation using cumulative dose limits

**DOI:** 10.1016/j.phro.2024.100547

**Published:** 2024-02-10

**Authors:** Sebastian Meyer, Lei Zhang, Yilin Liu, Li Cheng Kuo, Yu-Chi Hu, Yoshiya Yamada, Masoud Zarepisheh, Pengpeng Zhang, Laura Cerviño

**Affiliations:** a Department of Medical Physics, Memorial Sloan Kettering Cancer Center, New York, NY, United States; b Department of Radiation Oncology, Memorial Sloan Kettering Cancer Center, New York, NY, United States

**Keywords:** Re-irradiation, Spine SBRT, Automated planning

## Abstract

**Background and Purpose:**

The lack of dedicated tools in commercial planning systems currently restricts efficient review and planning for re-irradiation. The aim of this study was to develop an automated re-irradiation planning framework based on cumulative doses.

**Materials and Methods:**

We performed a retrospective study of 14 patients who received spine SBRT re-irradiation near a previously irradiated treatment site. A fully-automated workflow, DART (**D**ose **A**ccumulation-based **R**e-irradiation **T**ool), was implemented within Eclipse by leveraging a combination of a dose accumulation script and a proprietary automated optimization algorithm. First, we converted the prior treatment dose into equivalent dose in 2 Gy fractions (EQD2) and mapped it to the current anatomy, utilizing deformable image registration. Subsequently, the intersection of EQD2 isodose lines with relevant organs at risk defines a series of optimization structures. During plan optimization, the residual allowable dose at a specified tissue tolerance was treated as a hard constraint.

**Results:**

All DART plans met institutional physical and cumulative constraints and passed plan checks by qualified medical physicists. DART demonstrated significant improvements in target coverage over clinical plans, with an average increase in PTV D_99%_ and V_100%_ of 2.3 Gy [range −0.3–7.7 Gy] and 3.4 % [range −0.4 %−7.6 %] (p < 0.01, paired *t*-test), respectively. Moreover, high-dose spillage (>105 %) outside the PTV was reduced by up to 7 cm^3^. The homogeneity index for DART plans was improved by 19 % (p < 0.001).

**Conclusions:**

DART provides a powerful framework to achieve more tailored re-irradiation plans by accounting for dose distributions from the previous treatments. The superior plan quality could improve the therapeutic ratio for re-irradiation patients.

## Highlights

• Introduced a novel framework to optimize re-irradiation plans based on cumulative dose distributions.
• Clinically viable through its implementation in a commercial planning system via application program interface scripting.
• Demonstrated significantly superior plan quality compared to the current institutional approach.
• Offered a fully automated pathway that requires less than two hours for plan generation.

## 1 Introduction

Re-irradiation refers to a radiotherapy course that treats a previously irradiated volume or in which the cumulative dose to organs at risk (OARs) raises toxicity concerns [1]. Spinal malignancies are one key indication for re-irradiation, as around 40 % of cancer patients are developing spinal metastases [2], [3]. However, reirradiation in the spine can be particularly challenging, given the tissue tolerance of the proximal critical organs, such as the spinal cord and esophagus. Nowadays, stereotactic body radiation therapy (SBRT) is increasingly used in managing spinal tumors, including in the salvage re-irradiation setting [4], [5]. Compared to conventional radiation therapy, SBRT utilizes image-guided technology to deliver highly conformal ablative doses to the target in 1–5 fractions. The rapid dose falloff allows for better protection of the surrounding normal tissue. At our institution, we treated around 1500 spine SBRT plans in 2021, conservatively estimating that over 30 % were in a re-irradiation setting.

Re-irradiation planning remains a complex process that faces three significant challenges. First, radiobiological dose summation using equivalent dose in 2 Gy fractions (EQD2) is necessary to account for the differences in fraction schemes between prior radiation therapy (RT) and re-irradiation when computing cumulative OAR doses. Second, the substantial anatomical changes between treatment courses typically require deformable image registration (DIR) [6], which can introduce additional uncertainties. And third, expert consensus guidelines [7] and knowledge of cumulative tissue tolerance and recovery are limited [8], [9].

While re-irradiation has become more prevalent in recent years, only a few studies have investigated dedicated approaches for optimizing re-irradiation plans by accounting for the spatial dose distribution of the previous treatment [10], [11], [12], [13], [14], [15]. Existing solutions typically conservatively rely on rigid registration or the maximum OAR dose, are not fully streamlined for clinical application, or are only implemented in research software. The lack of dedicated tools and functionalities within commercial treatment planning systems (TPSs) currently restricts an efficient and effective workflow for re-irradiation planning [16].

This work introduces DART (Dose Accumulation-based Re-irradiation Tool), a fully automated planning framework that combines deformable dose mapping with a clinically viable planning strategy integrated within a commercial TPS. The goal was to improve the target coverage to maximize the therapeutic ratio while accounting for the spatial dose distribution of the prior RT and ensuring that plans meet cumulative OAR dose limits. We retrospectively applied our approach to spine SBRT re-irradiation and compared the results with the delivered plans that were optimized according to current institutional guidelines.

## 2 Materials and methods

### 2.1 Re-irradiation patient cohort

This retrospective planning study was performed under an institutionally approved protocol (IRB #16–422). We selected 14 patients who had received spinal SBRT re-irradiation to thoracic vertebral bodies (Table 1). The median time gap between treatments was 11 months (range 5–58 months). All patients had undergone a previous course of SBRT to a spinal segment in close proximity to the current treatment area. Patients were planned with nine posterior IMRT fields spanning over 160° (except for one case with 11 beams) and treated on Truebeam (Varian Medical Systems, Palo Alto, CA) machines using mainly 6X-FFF. Simulations were performed according to departmental spine SBRT procedures with patients undergoing a CT myelography, or an MRI (fused with the planning CT) for precise spinal cord delineation. The PTV was generated as a 0.2 cm expansion of the CTV and crops out the thecal sac.

**Table 1:** Characteristics of the patient cohort, listing the number of prior RT and ReRT cases for each group. The geometric overlap between prior RT and re-irradiation targets was quantified as partial (i.e., neighboring vertebral bodies), large (i.e., re-irradiation area contained prior RT target), or complete (i.e., same vertebral bodies treated). RT: radiation therapy, ReRT: re-irradiation.

	**Prior RT**	**ReRT**
**Target overlap**		
*Partial*	8	
*Large*	5	
*Complete*	1	
**Target size**		
*1*–*2 vertebral body*	9	7
*3*–*4 vertebral bodies*	5	5
*>4 vertebral bodies*	0	2
**Prescription**		
*9 Gy* × *3 fx*	7	2
*10 Gy* × *3 fx*	3	0
*6 Gy* × *5 fx*	2	1
*8 Gy* × *5 fx*	2	11

### 2.2 Current institutional practice for re-irradiation planning

For planning, the prior CT is rigidly registered with the re-irradiation planning CT, focusing on the current treatment area. Next, the field edge of the prior treatment in the superior-inferior direction is projected onto the current CT, and an additional margin of 5–10 mm is added to account for uncertainties in registration and setup. Finally, for each OAR, the segment that falls within the prior treatment field is considered as previously treated. A stricter predefined dose constraint is applied to each treated OAR compared to non-treated segments. For example, the spinal cord and esophagus D_max_ constraint for five fractions SBRT are reduced by 12 Gy and 25 Gy, respectively. The treatment plan optimization was performed using our proprietary automated expedited hierarchical optimization algorithm (ECHO) [17]. All constraints, objective functions, and parameters according to the clinical guidelines and priorities are loaded from the site-specific optimization template. The most important dose constraints for plan optimization, as well as more detailed information and evaluations of ECHO can be found in Hong et al. [18].

### 2.3 DART re-irradiation planning workflow

**Fig. 1:**
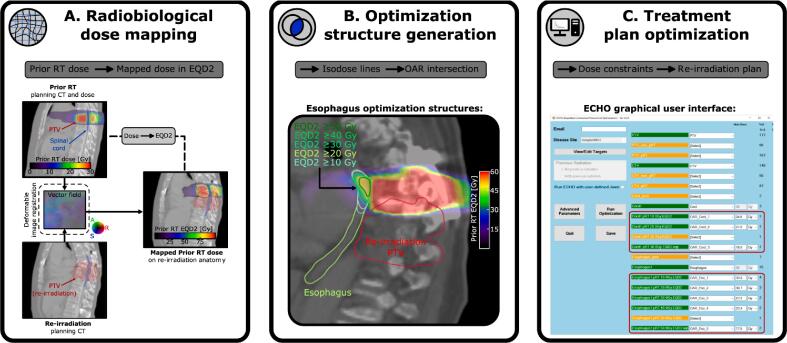
Dose Accumulation-based Re-irradiation Tool (DART). First, a radiobiological dose mapping is performed based on deformable image registration to calculate the prior RT dose distribution in terms of EQD2 within the re-irradiation anatomy. Next, the mapped EQD2 distribution derives a series of optimization structures for critical organs via Boolean operations. In the last step, the treatment plan is optimized using ECHO, where the structures are selected via a graphical user interface. Standard optimization objectives are already built in ECHO, and the user is also allowed to enter customized constraints for certain structures. The maximum dose constraints for the optimization structures are obtained as the remaining physical dose at the specific tissue tolerance. RT: radiation therapy, EQD2: equivalent dose in 2 Gy fractions, ECHO: expedited constrained hierarchical optimization.

The DART workflow is summarized in Fig. 1. It is fully automated and integrated within the TPS (Eclipse) using the scripting interface (ESAPI). Our approach consists of three main steps: (1) radiobiological dose mapping, (2) optimization structure generation, and (3) treatment plan optimization.

First, a DIR between both planning CTs was performed in Eclipse. Then, the physical dose distribution of the prior RT was mapped to the re-irradiation planning CT anatomy using the deformation vector field provided by the DIR and converted into EQD2, using the corresponding α/β ratio for each OAR (Fig. 1A). In this study, we focused on two critical organs that are of most importance in thoracic spine irradiation, the spinal cord and esophagus, and the α/β ratios used were 2 Gy and 3 Gy, respectively. Registration uncertainties were incorporated by applying a dose “smearing” during the dose mapping process. DIR is inherently associated with uncertainties, which result in a dosimetric uncertainty when the estimated transformation is used for dose mapping. To account for this effect, we do not directly map the dose from the homologous point identified by the DIR but instead, use the maximum dose found within a 0.2 cm spherical neighborhood around it. This provides a conservative estimation of OAR doses that considers the remaining local misalignment after DIR. Additionally, we restricted the dose smearing to be within the volume of the corresponding structure to avoid pulling the dose from nearby high-dose regions that do not belong to the specific OAR [19], [20].

Subsequently, a series of optimization structures was generated to consider the inhomogeneous prior RT dose distribution across the OARs during the plan optimization. To this end, the mapped dose distribution was automatically converted into isodose levels in steps of 10 GyEQD2. For both OARs and using high-resolution structures, the intersection with the isodose lines defined dedicated structures that represent the OAR portion that received a prior RT dose above the corresponding isodose level (Fig. 1B). An isotropic expansion of 0.2 cm was applied to structures below 1 cm^3^ but restricted to the original OAR volume to improve the robustness of the plan optimization.

DART treatment plan optimization was performed with ECHO. We created a dedicated template that integrates the previously generated optimization structures and represents them as maximum dose hard constraints within the optimization (Fig. 1C). Settings for all other structures were identical to the clinical paraspinal SBRT template. The physical dose constraint $D_{i}$ for OAR optimization structure i to achieve a given maximum cumulative OAR dose (i.e., the tissue tolerance; ${EQD2}^{\max}$) can be calculated according to the definition of EQD2 as

(1) $$D_{i}=n\times \left(\sqrt{{\left(\frac{\alpha /\beta }{2}\right)}^{2}+\frac{\left({EQD2}^{\max}-{EQD2}_{i}^{\mathrm{pRT}}\right)}{n}\left(2Gy+\alpha /\beta \right)}-\frac{\alpha /\beta }{2}\right)$$

where n is the number of fractions in the re-irradiation plan. ${EQD2}_{i}^{\mathrm{pRT}}$ is the maximum relevant prior RT dose within the optimization structure, considering that the overlapping structures are automatically segregated within ECHO. To ensure a fair comparison, the maximum cumulative dose to each OAR for the clinical plans was assumed as the tissue tolerance ${EQD2}^{\max}$ in Eq. (1) and the same beam configurations were used. Hence, we evaluate the benefit of DART due to the capability of considering the spatial distribution of the pRT dose without increasing cumulative OAR doses compared to the delivered clinical plans. Cumulative dose distributions were calculated as a radiobiological dose summation, that is, individually converting the mapped prior-RT and re-irradiation dose distributions into EQD2 and adding them on the voxel level.

### 2.4 Plan evaluation and comparison

Clinical and DART re-irradiation plans were compared in terms of target dose (D_99%_ and D_95%_), coverage (V_95%_ and V_100%_), high-dose spillage (>105 %) outside the PTV, and the relevant institutional OAR criteria (esophagus $D_{\max}$ and $D_{{5cm}^{3}}$; spinal cord $D_{\max}$ and $D_{{0.035cm}^{3}}$). In addition, the plan quality was assessed using the plans’ duty cycle, Paddick’s conformity index [21], ICRU gradient index, and homogeneity index [22]. We also evaluated the cumulative dose distributions to validate that DART plans did not exceed the maximum OAR doses. Statistical comparisons were performed using paired t-tests. Finally, we evaluated the robustness of the plans against DIR uncertainty by varying the radius for the dose smearing when calculating the cumulative dose distributions.

## 3 Results

**Fig. 2:**
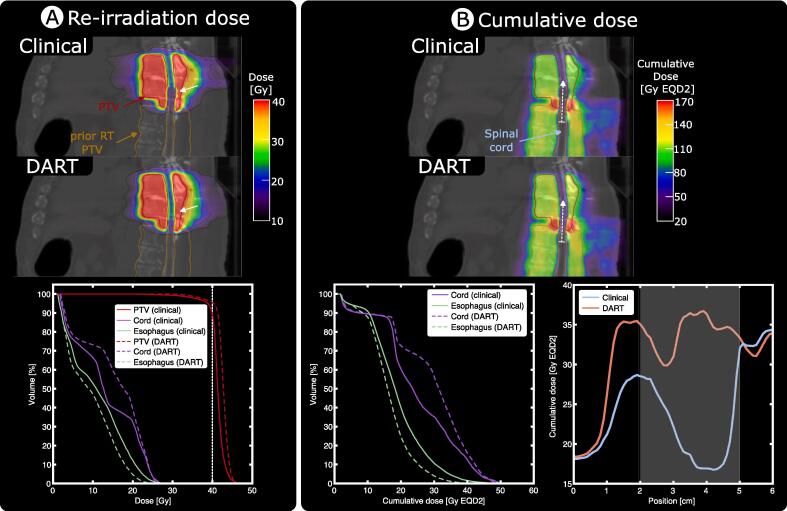
Dose distributions for an example case. Comparison of the clinical and DART plan for a patient treated at the T7-T9 vertebral bodies (8 Gy × 5) after receiving prior RT at the T10-T12 vertebrae (8 Gy × 5). (A) Physical dose distribution and corresponding PTV DVH of the re-irradiation plans. (B) Cumulative dose distributions in equivalent dose in 2 Gy fractions (EQD2) and corresponding DVHs for relevant organs at risk. The line profile corresponds to the dashed white arrow along the spinal cord shown on top of the dose distribution. RT: radiation therapy, DVH: dose-volume histogram, DART: Dose Accumulation-based Re-irradiation Tool.

A plan comparison for an example patient is shown in Fig. 2. DART provided superior PTV coverage (Fig. 2A, white arrow) with a 2.8 Gy (9 %) higher D_99%_ and 6.4 % higher V_100%_, while fulfilling all institutional OAR dose constraints. Cumulative dose distributions and corresponding dose-volume histogram (DVHs) (Fig. 2 B) demonstrate that DART did not increase OAR D_max_ in terms of EQD2 compared to the clinical plans, while only resulting in a larger volume receiving a spinal cord dose between the intermediate 20 and 40 GyEQD2. By spatially adjusting the re-irradiation dose limits based on the prior dose, we were able to avoid regions of too stringent OAR constraints that negatively impacted coverage of the surrounding PTV (Fig. 2B, profiles).

**Fig. 3:**
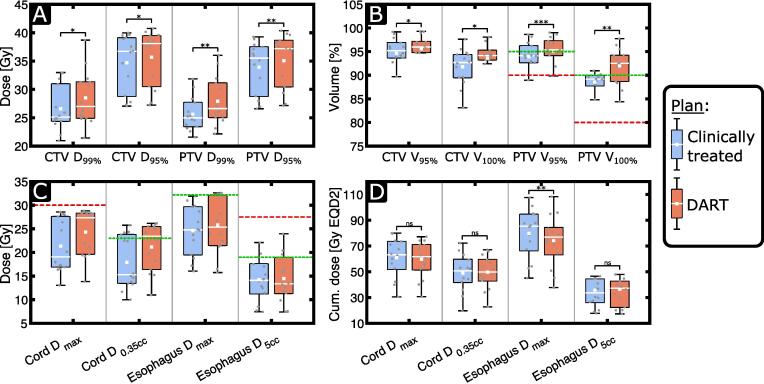
Dose metric comparison for the patient cohort. Re-irradiation plan (A and B) target coverage and (C) critical structure dose. Ideal and acceptable criteria according to institutional policies for five fraction treatments are indicated by the dotted green and dashed red lines, respectively. (D) Cumulative doses for critical structures. The mean and median of the distribution are represented by the central white line and marker, respectively, the box corresponds to the first and third quartile, and each whisker indicates 1.5 times the interquartile range. Results of the paired *t*-test are indicated by ns, *p<0.05, ** p<0.01, and ***p<0.001. CTV: clinical target volume, PTV: planning target volume, DART: Dose Accumulation-based Re-irradiation Tool, ns: not significant. (For interpretation of the references to colour in this figure legend, the reader is referred to the web version of this article.)

The comparison of both approaches is presented in Fig. 3. All DART plans passed rigorous plan checks by qualified medical physicists. DART plans provided significantly superior target coverage (Fig. 3A and B), with a mean improvement in PTV D_99%_ and V_100%_ of 2.3 Gy [range −0.3–7.7 Gy] and 3.4 % [range −0.4 %−7.6 %], respectively. The corresponding results for the CTV were 1.9 Gy [range −1.6–6.8 Gy] and 1.9 % [range −1.0 %−5.5 %]. An increase of up to 3.1 Gy in D_95%_ was observed for CTV and PTV. In addition, high-dose spillage (>105 %) was reduced by 1 ± 2 cm^3^ [range −2–7 cm^3^]. The relevant clinical dose limits (Fig. 3C) were achieved for all critical structures in both approaches. At comparable cumulative spinal cord D_max_, a reduction of up to 16.2 GyEQD2_3Gy_ in esophagus maximum dose was observed for DART (Fig. 3D).

**Fig. 4:**
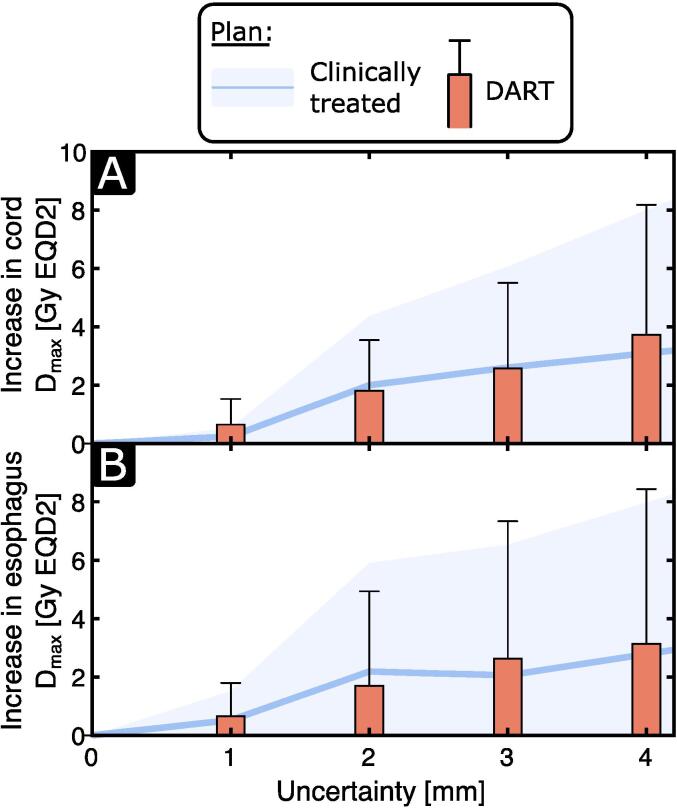
Analysis of plan robustness to registration uncertainty within a clinically relevant range. Increase in cumulative (A) spinal cord and (B) esophagus maximum dose for increasing uncertainty (that is, isotropic dose smearing radius) relative to 0 mm. Mean and standard deviation are shown for the clinically treated (blue) and DART (orange) plans. (For interpretation of the references to colour in this figure legend, the reader is referred to the web version of this article.)

The plan quality metric comparison summarized in Table 2 shows that DART achieved substantially superior plans. The average improvements in conformity, homogeneity, and gradient index compared to the clinical plans were 3.3 % [range −3.5 %−16.7 %], 19.4 % [range 0 %−37.4 %], and 5.0 % [range −8.4 %−16.5 %], respectively. Fig. 4 shows the impact of increasing DIR uncertainties on the spinal cord and esophagus D_max_ when computing the cumulative dose distribution. Overall, both approaches exhibit a similar sensitivity, with an average increase of less than 5 GyEQD2 for an uncertainty of 4 mm.

**Table 2:** Plan quality metric comparison for the patient cohort. Results are quantified by the mean ± standard deviation over the complete patient cohort. The p-value refers to the *t*-test evaluation. DART: Dose Accumulation-based Re-irradiation Tool, ns: not significant.

Metric	Clinically treated	DART	p
Conformity index	0.78 ± 0.08	0.80 ± 0.07	<0.05
Homogeneity index	0.37 ± 0.12	0.31 ± 0.13	<0.001
Gradient index	5.05 ± 0.97	4.79 ± 0.97	<0.05
Duty cycle	8.44 ± 1.34	7.86 ± 0.87	ns

## 4 Discussion

This work introduced DART, an automated planning approach that uses scripting to build upon existing software infrastructure within a commercial TPS. We retrospectively applied this approach to 14 paraspinal SBRT re-irradiation cases and were able to achieve statistically significant improvements in plan quality compared to the current clinical procedure at our institution.

The results of this proof-of-concept study demonstrate the feasibility of efficiently optimizing re-irradiation plans based on cumulative tissue tolerances and with uncertainty taken into consideration as well. We were able to achieve significant improvements in overall treatment plan quality by spatially adjusting the OAR re-irradiation constraints, resulting in slightly higher volumes receiving an intermediate dose while still meeting the D_max_ limit. Studies indicated that target D_min_ and D_95%_ can be important factors for local control [23], [24]; hence, DART has the potential to improve the therapeutic ratio in spine SBRT re-irradiation. This is attributed to the capability of DART to consider the spatial distribution of the prior RT dose within the re-irradiation optimization instead of conservatively making a binary separation as in the current clinical workflow.

Our approach was fully automated with a computational time of less than 2 h, while most plans were completed within 1 h, depending on server usage. Most time was spent on plan optimization (54.4 ± 28.2 min), while dose mapping and structure generation took only 2.3 ± 0.7 min and 3.9 ± 0.8 min, respectively. This workflow is substantially more efficient than the current manual planning, including a special medical physics consult, which can require up to 10 h alone [10].

Detailed data on cumulative tolerances for normal tissues are lacking, and only limited guidelines are available in the literature [25]. For example, Doi et al. [9] did not observe any radiation myelopathy for cumulative spinal cord doses of more than 100 GyEQD2, whereas Saghal et al. [26] recommended a thecal sac D_max_ limit of 70 GyEQD2. In addition, a time-dependent recovery factor should be considered when calculating the re-irradiation tolerance [10]. In this study, we imposed the same cumulative OAR dose limit for the DART plan as observed within the clinical plan for each patient to ensure a fair comparison. However, DART plans could also be designed to allow cumulative OAR doses up to the aforementioned tissue tolerances. This has the potential to further enhance the re-irradiation plan quality as the clinical approach can not directly consider a given tissue tolerance during the optimization. DART also provides the functionalities to consistently incorporate DIR-based cumulative doses into clinical workflows, which is especially relevant for cases like curved T-spines and patients with significant anatomical changes. The carefully accumulated dose can be highly valuable for further studies on tissue tolerances.

Our proposed workflow is efficient and effective but faces a few limitations. First, dose mapping and accumulation are affected by registration uncertainties [27]. Validation of DIR is highly challenging as the ground truth is almost never available, and one often must rely on indirect measures such as surrogates (e.g., contour overlap measures) or DIR confidence (e.g., uncertainty or plausibility). To this end, we have incorporated a 0.2 cm dose smearing that conservatively accounts for uncertainties associated with registration and individual contours. Moreover, the long time between prior and re-irradiation treatments can result in complex anatomical changes that can violate the assumption of a one-to-one anatomical mapping and the energy/mass conservation during dose resampling [28]. Advanced research algorithms that employ diffeomorphic transformations could help to overcome some of these challenges by guaranteeing numerically and physiologically realistic deformations. Our current DART workflow does not restrict the type of registration algorithm and allows the importation of external deformation vector fields in various formats, and future work will evaluate the impact of DIR on the plan quality. Our D_max_ sensitivity analysis found an average increase of less than 5 GyEQD2, which is relatively low compared to potential tissue tolerances of around 100 GyEQD2 [9], [29].

Finally, some cases required multiple optimizations with small manual adjustments of the dose limits to achieve the desired cumulative tolerance, and we accepted a deviation of 1 GyEQD2 with respect to the optimization goal. The reason is that the leaf sequencing and final dose calculation after the ECHO optimization must be performed using the FDA-approved dose engine in Eclipse [17], which can lead to small discrepancies in D_max,_ especially for small structures. We aim to address this in future studies by exploring variable EQD2 steps in the optimization structure generation. Nevertheless, DART provides a more efficient solution than the current labor-intensive and iterative clinical workflow.

Most re-irradiation planning approaches described in the literature are incompatible with clinical requirements. However, Murray et al. [15] recently presented the first viable solution by integrating a complete re-irradiation planning pathway in a research version of a commercial TPS. They used an EQD2-based biological optimization that incorporates the prior treatment as background dose, while target objects are formulated as physical doses in the re-irradiation plan only. Their approach generated clinically acceptable plans and yielded improved plan quality in several cases. In contrast, our approach only used physical dose optimization based on prior RT isodose structures [14], providing two main advantages. First, simultaneously considering cumulative (biological) OAR limits and re-irradiation (physical) dose target constraints can become very challenging for a planner to iteratively modify the optimization parameters. Second, such composite optimization restricts the use of established methods for automated planning that can improve efficiency and plan quality. However, one disadvantage of our methodology is the current inability to include volumetric cumulative dose constraints. While additional work is necessary to improve our planning framework, it is important to note that data on cumulative tissue tolerances is mainly expressed as maximum or near-maximum dose.

In future work, we intend to investigate potential modifications of the ECHO template (e.g., the objectives and plan normalization), which was based on the current institutional paraspinal settings. Tailoring it to re-irradiation applications could translate into further improved target coverage. In addition, we aim to extend our dose-smearing approach to account for the spatial variation of DIR uncertainty, that is, using a spatially-dependent smearing radius. Moreover, we need to consider that the DIR uncertainty is typically not isotropic due to the underlying parametrization of the geometric transformation (e.g., via B-Splines).

In conclusion, DART offers a versatile framework for creating a personalized re-irradiation plan that considers the patient's previously received dose distribution. This enabled a significantly better plan quality at the same tissue tolerance, which could potentially translate into an improved therapeutic ratio for patients with recurrent spinal tumors.

## CRediT authorship contribution statement

**Sebastian Meyer:** Methodology, Writing – original draft, Visualization, Formal analysis. **Lei Zhang:** Data curation, Writing – review & editing. **Yilin Liu:** Validation, Writing – review & editing. **Li Cheng Kuo:** Methodology. **Yu-Chi Hu:** Software. **Yoshiya Yamada:** Data curation, Resources. **Masoud Zarepisheh:** Software, Writing – review & editing. **Pengpeng Zhang:** Supervision, Conceptualization, Writing – review & editing. **Laura Cerviño:** Supervision, Writing – review & editing.

## Declaration of Competing Interest

The authors declare that they have no known competing financial interests or personal relationships that could have appeared to influence the work reported in this paper.
